# Metabolic Evaluation in Children aged 3 months to 2 years with Global Developmental Delay

**DOI:** 10.1007/s12098-023-04927-9

**Published:** 2023-12-12

**Authors:** Rochelle Natasha Gomes, Ramesh Bhat Y, Sandesh Kini, Pushpa G Kini, A Shrikiran, CM Suneel

**Affiliations:** Department of Pediatrics, Kasturba Medical College, Manipal, Manipal Academy of Higher Education, Manipal, India

**Keywords:** Global developmental delay, Children, Inborn errors of metabolism

## Abstract

**Objectives:**

To study the clinical profile and role of metabolic evaluation in children aged 3 mo to 2 y with global developmental delay (GDD) of unclear etiology.

**Methods:**

In this prospective study, demographic and clinical data along with first line metabolic test results [blood glucose, arterial blood sample analysis, renal function tests, uric acid, serum electrolytes, liver function tests (LFTs), plasma ammonia, arterial blood lactate and pyruvate, urine ketone/ reducing substances] were documented and analyzed. Tandem Mass Spectroscopy (TMS) and Gas Chromatography and Mass Spectrometry (GC-MS) data were also analysed.

**Results:**

Of 101 eligible children, 48 were excluded. Among 53 children included in the study, 32 (60.3%) were less than 1 y and 21 (39.7%) were more than 1 y. Four major developmental domains were almost equally affected in 16 (30.1%), three domains in 4 (7.5%) and two domains in 33 (62.4%) children. Fourteen (26.4%) children were found to have a probable metabolic disorder based on initial tests- 10 mitochondrial disorders, 3 organic-acidemias and 1 fatty-acid-oxidation defect. Further, on TMS and GC-MS tests, 11 (20.7%) had a metabolic disorder- 7 mitochondriopathies, 2 methylmalonic-aciduria, 1 each with glutaric-acidemia and ethylmalonic-aciduria.

**Conclusions:**

Among children with GDD of unclear etiology, metabolic errors constitute a small proportion of etiology. In this group early metabolic tests could identify potentially treatable conditions.

## Introduction

Global developmental delay (GDD) affects 3–10% of children below 5 y of age [1–5]. The evaluation of GDD in preschool children varies across centres and pediatricians [2, 6–9]. Metabolic causes constitute one of the causes for GDD that are treatable. The contribution of metabolic etiology to global developmental delay in different studies range from 1–65% [1, 2, 10, 11].

Metabolic disorders lead to accumulation of substrates resulting in a spectrum of minor to severe neuro-psychiatric manifestations that often lead to lifelong disability or death [3]. Developmental delay may be one of the presenting complaints of inborn errors of metabolism [4]. Development is an orderly process, thus, it is important, to identify the sequence of attainment of milestones, and delays [12]. Rapid enlargement of the brain occurs in the late months of fetal and early postnatal life - by birth the structure of the brain is complete, with head size around 70% of the expected adult head size, this reaches 90% of the adult head size by 2 y of age. Hence, first 2 y of life are the crucial period for brain development, during which maximum neuromotor functions and cognitive abilities are attained [12]. Early recognition of GDD and initiation of interventions in the form of stimulation, treatment of associated functional impairments, physiotherapy and rehabilitation prevent progression of complications and improves neurological outcomes [12–15].

The history and physical examination may provide up to 40% of etiologic diagnosis in children with GDD and in the rest etiology remains often unclear [1, 5, 16, 17]. If a metabolic disorder is suspected clinically, there should be a low threshold for performing metabolic tests as some inborn errors of metabolism present in a very subtle manner and are easy-to-miss on screening tests. Selective and targeted tests should be done as per recommended guidelines [6]. Tier-1 or general metabolic tests for inborn errors of metabolism (IEMs) in children with unexplained GDD have been recommended even with absent clinical red flags or a negative newborn screen [5, 6]. The algorithms devised by the ‘American Academy of Pediatrics’ (AAP), the ‘American Academy of Neurology’ (AAN) and the ‘Treatable Intellectual Disability Endeavor’ (TIDE) protocol recommend these first tier tests [1, 2, 6, 18]. In this context, the present study was undertaken to evaluate the clinical profile and role of metabolic evaluation in children aged 3 mo to 2 y with GDD of unclear etiology.

## Material and Methods

This prospective observational study was conducted in the Department of Pediatrics of a Medical college hospital.

Children aged 3 mo to 2 y, admitted in the Department of Pediatrics between March 2021 and September 2022, with unexplained GDD (one or more of the following: mild encephalopathy with or without infrequent seizures, hypotonia/hypertonia, prolonged feeding time, failure to thrive, unexplained mild respiratory distress or infrequent vomiting) were enrolled in this study. Children with history of transient hypoglycemia in newborn period or at presentation were also included. The study was time bound and included all the children fulfilling the inclusion criteria in the study period. Such children were admitted for a day or two for further evaluation, evaluation of comorbidity particularly hearing and vision assessment and to initiate specific therapy if needed. These children are mostly from distant places. During the admission, parents were taught physiotherapy and or occupational therapy as needed. Children with identified causes of GDD such as perinatal asphyxia/hypoxic ischemic encephalopathy (HIE) stage 2 or 3, TORCH infections, post-meningitis sequelae, chromosomal disorder or malformations, hypothyroidism, bilirubin encephalopathy, neuroregression and children who were already on megadoses of vitamin supplements were excluded. The study did not include children with acute presentation of encephalopathy, severe acidotic breathing, persistent frequent vomiting or acute unresponsive state.

Institutional Ethics Committee clearance (IEC- 859/2020) was obtained and CTRI registration (CTRI/2021/04/032521) was done. Informed consents were obtained from the parents of the study children.

In each case, history and examination findings were recorded in the predesigned study proforma. All children underwent initial metabolic studies including blood glucose, arterial blood sample analysis, renal function tests, uric acid, serum electrolytes, liver function tests (LFTs), plasma ammonia, arterial blood lactate and pyruvate, and urine ketone/ reducing substances. Tandem Mass Spectroscopy (TMS) and Gas Chromatography and Mass Spectrometry (GC-MS) tests when indicated were obtained and analysed. Electroencephalography (EEG), neuroimaging, ophthalmological and hearing evaluation, and thyroid function tests were carried out based on clinical indications.

Initial metabolic tests were analyzed as follows: Hypoglycemia: Serum glucose less than 60 mg/dL, elevated lactate: more than 22 mg/dL, hyperammonemia: serum ammonia more than 150 mcg/dL, low urea: urea less than 5 mg/dL, elevated lactate by pyruvate ratio: more than 50, metabolic acidosis: pH <7.35 with bicarbonate <18 mEq/L, high anion gap (AG): >16 and elevated Creatine phosphokinase (CPK): >260 U/L. Total bilirubin >2 mg/dL, direct bilirubin >1 mg/dL with elevated transaminases thrice the normal (normal AST and ALT up to 40 IU/L) were considered suggestive of hepatic involvement, urine ketones +/- were considered for ketotic and non-ketotic hypoglycemia and urine reducing substances- presence/absence was considered.

From the abnormalities in the initial metabolic workup, following probable metabolic diagnosis were considered for the purpose of analysis: (1) Urea cycle disorders- hyperammonemia, low urea, with or without liver function test abnormalities, (2) Fatty acid oxidation defect disorders- metabolic acidosis, hypoglycemia, absent urinary ketones, (3) Organic academia- acidosis, increased AG, increased ammonia >150 mcg/dL, hypoglycemia, (4) Galactosemia-hypoglycemia with urinary reducing substances, (5) Mitochondrial disorders- increased lactate or increased L/P ratio >50, abnormalities in LFT, increased CPK and (6) Glycogen storage disorders- hypoglycemia, abnormal liver function tests, raised CPK and ketonuria.

Specific abnormalities found in TMS, GC-MS were categorized as normal or as one of the above disorders.

Statistical analysis was done using SPSS software version 24. Descriptive analysis including frequency distribution and percentages for categorical data were used for analysis. There were no drop outs during the study.

## Results

**Fig. 1:**
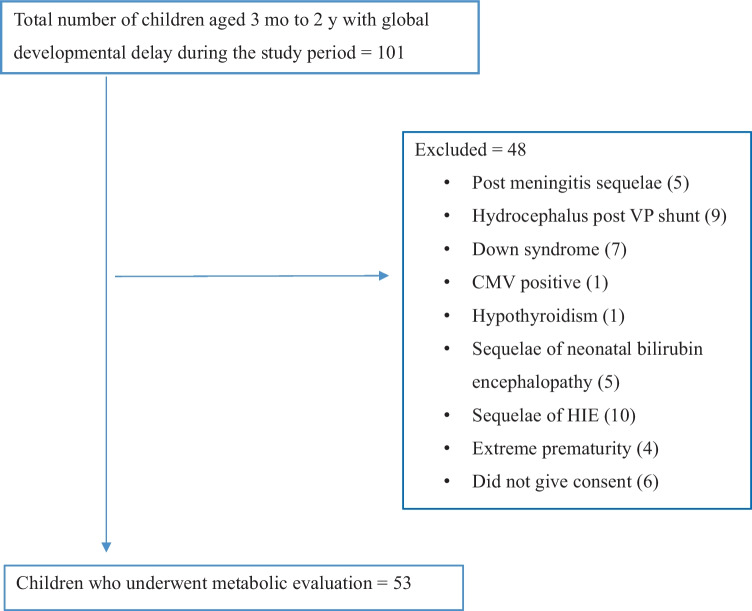
Study flow diagram. *CMV* Cytomegalovirus, *HIE* Hypoxic ischemic encephalopathy, *VP* Ventriculo-peritoneal

The study flow diagram is presented in Fig. 1. The total number of children fulfilling the inclusion criteria during the study period was 101. After exclusion, 53 children were further analyzed.

The demographic data of the study participants is presented in Table 1. Thirty-two (60.3%) children were less than 1 y and 21 (39.7%) were more than 1 y of age. The male: female ratio was 2.5:1. Parental consanguinity was present in 21 (39.6%). Of 53 children, 49 (92.4%) were born at term gestation. One (1.8%) child’s sibling was affected with propionic acidemia.

**Table 1:** Demographic characteristics of children with GDD of unclear etiology (n = 53)

Parameter	n (%)
Age	
3–6 mo	15 (28.3)
7–12 mo	17 (32.1)
13–18 mo	10 (18.9)
19–24 mo	11 (20.7)
Gender	
Male	38 (71.7)
Female	15 (28.3)
Parental consanguinity	
Present	21 (39.6)
Absent	32 (84.4)
Gestation at birth	
Term	49 (92.4)
Preterm	4 (7.6)
History of neonatal hypoglycemia (transient)	
Present	5 (9.4)
Absent	48 (90.6)
Children with development delay in the family	
Present	12 (22.6)
Absent	41 (77.4)

*GDD* Global developmental delay

Symptoms at presentation included feeding difficulties in 42 (79%), convulsions and tone abnormalities in 24 (45.2%) each, hurried breathing, vomiting, lethargy and altered sensorium in 5 (7.6%) each. Developmental delay was predominantly fine motor in 12 (22.6%), gross motor in 11 (20.7%), speech and language in 9 (16.9%), personal/social in 5 (9.4%) children. Four major domains were almost equally affected in 16 (30.1%) children. Absent neck-control was the most common presenting symptom in less than one-year-old children and speech delay in more than one-year-old children. Twenty-four (45.2%) children had associated seizures- generalized tonic-clonic seizure (GTCS) in 13 (24.5%), myoclonic in 10 (18.8%) and focal in one (1.8%).

Examination findings included hypertonia and exaggerated deep tendon reflexes (DTRs) in 22 (41.5%) each, hepatomegaly in 9 (16.9%), focal neurological deficits in 6 (11.3%), tachypnea and hypotonia in 5 (9.4%) childen each. Microcephaly was present in 13 (24.5%) children. Dysmorphic features included telecanthus in 5 (9.4%) children, long philtrum in 2 (3.8%) children, mid facial hypoplasia in 4 (7.5%) children and low set ears in 2 (3.8%) children. Neurocutaneous markers included insignificant café au lait spots in 12 (22.6%) children and single ash leaf macule in one (1.9%) child.

Low urea (2 mg/dl) was present in one (1.8%) child, however it was not associated with hyperammonemia. Metabolic acidosis was present in 13 (24.5%) children, 11 (20.7%) had associated high anion gap. Urinary ketones were present in 4 (9.3%) children. Hypoglycemia was present in 2 (3.7%) children, with absent urinary ketones. Elevated lactate was present in 14 (26.4%) children. The lactate/pyruvate ratio was elevated in 10 (18.8%) children. CPK was elevated in 5 (9.4%) children. Initial metabolic tests were suggestive of IEM in 14 (26.4%) children- 10 (18.8%) mitochondrial disorders, 3 (5.6%) organic acidemia and one (1.8%) fatty acid oxidation defect.

TMS and GC-MS tests could be obtained only in 25 children due to financial constraints. Eleven (20.7%) were found to have a metabolic disorder. Acyl carnitine was high in 2 children, total carnitine was low in 1 child, and free carnitine was low in 1 child. They had associated features suggestive of mitochondrial disorders. One child had low free carnitine with elevated C5DC/C2 ratio suggestive of glutaric aciduria type 1 (GA-1), one child had elevated levels of total and free carnitine with increased C0/C16+18 ratio suggestive of CPT1 deficiency and 5 children had elevation of multiple aminoacids suggestive of mitochondriopathy. On urine GC-MS, 7 children had elevated levels of lactic acid, pyruvic acid, ketones, fumaric acid and dicarboxylates- suggesting mitochondriopathy, one child had grossly elevated lactic acid and 3 hydroxyglutaric acid, suggestive of glutaric acidemia type 1, two children had methylmalonic aciduria suggestive of vitamin B12 deficiency or dependency and one child had ethylmalonic aciduria. The correlation between initial and final metabolic workup in the study participants is presented in Table 2. The final metabolic work up was positive in 7 out of 11 children in whom initial tests were suggestive of a metabolic disorder. Final metabolic work up was positive in additional 4 cases in whom there were no clue from initial metabolic work up.

**Table 2:** Correlation between initial and final metabolic workup in children with GDD of unclear etiology (n = 25)

	Final metabolic workup	
	Suggestive of metabolic disorder	Not suggestive of metabolic disorder
Initial metabolic workup		
Suggestive of probable metabolic disorder (n = 11)	7	4
Not suggestive of probable metabolic disorder (n = 14)	4	10

*GDD* Global developmental delay

Hearing loss was present in 6 (14.2%) children (sensorineural type). Vision abnormalities were present in 12 (5.9%) children- cortical blindness in one (8.3%) child, optic atrophy in one (8.3%) child and refractive errors in 10 (83.4%) children. MRI brain findings included frontotemporal atrophy with subdural hygroma in 7 (28%) children, benign enlargement of subarachnoid space (BESS) in 2 (8%) children, partial corpus callosal agenesis in 3 (12%) children, and calcifications in right frontal region in 1 (4%) child, periventricular leukomalacia in 4 (16%) children and hyperintensities in globus pallidus in 1 (4%) child. Abnormal EEG findings included epileptiform encephalopathy in 3 (15%) children, encephalopathic changes in 4 (20%) children, focal epilepsy in 6 (30%) children, generalized spike and wave discharges in 1 (5%) child and multifocal epilepsy in 3 (15%) children.

Few interesting study findings and laboratory correlation are presented in Table 3. Study participant 2 had associated left hemiparesis and impaired vision. Initial metabolic tests were normal. MRI brain and angiography showed encephalomalacia with gliotic changes and cyst formation in the right frontotemporal region with mild dilatation of the right lateral ventricle and attenuated right middle cerebral artery. Study participant 38 had associated infantile spasms, sustained exaggerated DTRs and ankle clonus, brain imaging showed periventricular hyperintensities, EEG showed right temporal epilepsy. The child had normal initial metabolic tests, vision and hearing. Study participant 45, born of second degree consanguineous marriage, presented with infantile spasms, polymorphic seizures, spasticity and exaggerated DTRs; initial metabolic tests showed hyperammonemia.

**Table 3:** Clinical and laboratory correlation in children with GDD of unclear etiology

Serial no.	Age in months and sex	Clinical profile	Initial metabolic screen	Final metabolic screen (TMS and GC-MS)	Other relevant investigations
6	12 mo, male	Absent neck control, flexor myoclonus, spasticity, impaired vision and hearing, obesity, microcephaly	Mitochondrial disorder	Not done	EEG- multifocal epileptiform abnormalities, hearing- bilateral TEOAEs absent, MRI Brain- mild ventriculomegaly
9	5.5 mo, male	Absent neck control, sparse brown hair, GTCS, encephalopathy, chronic malnutrition, hypertonia	Mitochondrial disorder	Normal	MRI Brain- cerebral atrophy with subdural hygroma. Maternal HIV test was negative, newborn screen for biotinidase deficiency was negative.
10	12 mo, male	Inability to stand without support, speech delay, scalp alopecia, chronic malnutrition, absent dentition, hypotonia	Normal	Increased 3 hydroxybutyrate and acetoacetate s/o organic aciduria	Myopia
11	4 mo, male	Impaired vision, no neck control, 3rd degree consanguinity, extensor myoclonic jerks, rotatory nystagmus, spasticity, brisk DTRs	Normal	Normal	VEP- no latency in both eyes, MRI- BESS, frontotemporal atrophy, ophthalmology- delayed visual maturation
12	12 mo, male	Inability to sit without support, extensor myoclonus, microcephaly, spasticity	Normal	Normal	MRI Brain- bilateral parieto-occipital hyperintensities with mild hygroma, EEG- Right temporal lobe epilepsy
14	12 mo, male	Inability to hold neck, failure to thrive, second degree parental consanguinity, h/o previous neonatal death, microcephaly, hepatomegaly, flexor myoclonus and focal clonic seizures, spastic quadriparesis	Normal	Normal	EEG- normal, MRI brain-normal, CDG Type 1g, AR inheritance, in homozygous state, on ALG12 NM_024105.4 on exon 10, variant C.1288a>Cp. (THr430Pro)
16	7 mo, female	Inability to hold neck, speech delay, recurrent respiratory infections, generalized hyperpigmentation, second degree consanguinity, microcephaly, hepatomegaly, hypotonia, tachypnea	Mitochondrial disorder	Elevated adipic, suberic, 2 oxoglutaric acid and a small peak of ethylmalonic acid, suggestive of Riboflavin deficiency	
23	4 mo, male	Absent neck control, speech delay, hurried breathing, poor activity, vomiting	Mitochondrial disorder	Elevated lactate, pyruvate, 3 hydroxybutyrate, acetoacetate, fumarate, adipic acid and suberic acid suggestive of mitochondriopathy	Echocardiography- severe PAH
24	4 mo, male	Increased head circumference, absent neck control, poor weight gain, second degree parental consanguinity, low set ears, post axial polydactyly	Organic acidemia	Low free carnitine, elevated C5DC and C5DC/ C2 ratio, grossly elevated glutaric acid and 3 hydroxyglutaric acid suggestive of glutaric acidemia type 1	MRI brain- prominent CSF spaces along the frontal, anterior temporal, anterior hemispheric fissure and sylvian fissures
25	4 mo, male	Absent neck control, tachypnea	Mitochondrial disorder	Elevated alanine, leucine-isoleucine, valine, methionine, grossly elevated lactate, pyruvate, fumarate, succinate and glutarate suggestive of mitochondriopathy affecting liver, most probably DNA depletion syndrome.	Echocardiography- severe PAH
26	6 mo, female	Absent neck control, uncontrollable GTCS, third degree parental consanguinity, chronic malnutrition, hypotonia	Normal	Elevated total and free carnitine with increased C0/C16 + 18 ratio suggestive of CPT1 deficiency	MRI Brain- normal, EEG- focal epilepsy
27	12 mo, male	Absent neck control, speech delay, poor weight gain, chronic malnutrition	Mitochondrial disorder	Low free/ acyl ratio, elevated alanine, elevated lactate, and ketones- 3 hydroxybutyrate, 3 hydroxyisovalerate suggestive of mitochondriopathy	
28	7 mo, male	Absent neck control, speech delay	Mitochondrial disorder	Normal	USG abdomen- horseshoe kidney
30	7 mo, male	Absent neck control, GTCS, second degree parental consanguinity, inconsolable cry, refusal to feed, hypotonia	Fatty acid oxidation defect	Elevated lactate, pyruvate and fumarate suggestive of mitochondriopathy	BERA- no peak 5 even at 70 dBnHL, EEG normal, MRI Brain- bilateral symmetrical T2W flair hyperintensity involving globus pallidus
34	4 mo, male	Absent neck control, GTCS	Mitochondrial disorder	Elevated lactate with significantly elevated 4- hydroxyphenylacetate, 4-hydroxyphenylpyruvate suggestive of mitochondriopathy affecting liver	EEG- normal
36	7 mo, male	Absent neck control, myoclonic jerks	Organic acidemia	Decreased low free/acyl ratio, elevated alanine, glycine with moderately elevated isoleucine, leucine, valine, methionine and phenylalanine, elevated lactate, pyruvate and ketones (3 hydroxybutyrate and acetoacetate) suggestive of mitochondriopathy	EEG- multifocal epileptiform abnormalities
40	6 mo, male	Absent neck control, history of prior intrauterine death, IEM in elder sibling	Mitochondrial disorder	Elevated lactate and succinate suggestive of mitochondriopathy	
41	6 mo, male	No roll over or neck control, infantile spasms, visual impairment	Mitochondrial disorder	Normal	EEG- generalized epilepsy, MRI Brain- frontotemporal atrophy, ophthalmology- compound myopic astigmatism
53	12 mo, male	Absent neck control, speech delay, poor feeding, vomiting	Organic acidemia	Methylmalonic acidemia suggestive of vitamin B12 deficiency	

*AR* Autosomal recessive, *BERA* Brainstem evoked response audiometry, *BESS* Benign enlargement of subarachnoid space, *CDG* Congenital disorder of glycosylation, *CSF* Cerebrospinal fluid, *DTR* Deep tendon reflexes, *EEG* Electroencephalography, *GC-MS* Gas chromatography mass spectroscopy, *GDD* Global developmental delay, *GTCS* Generalized tonic clonic seizures, *MRI* Magnetic resonance imaging, *PAH* Pulmonary arterial hypertension, *TEOAE* Transient evoked otoacoustic emissions, *TMS* Tandem mass spectroscopy, *USG* Ultrasonography, *VEP* Visual evoked potential

## Discussion

Children with developmental delay cause anxiety in parents and sometime remain as a challenge as no etiology could be found after thorough history and clinical examinations. The present study aimed to find out the role of metabolic tests in these children with GDD of unclear etiology. Neurological development is almost complete by 2 y of age. Hence, there is a need to identify developmental delay and possible etiology in early life so that appropriate treatment and early intervention could be instituted. Several recommendations have been formed to evaluate metabolic diseases presenting with GDD [1, 2, 6, 10]. The present study is a well conducted prospective observational study, reported in accordance with ‘The Strengthening the Reporting of Observational Studies in Epidemiology' (STROBE) Statement.

The male predominance in the present study sample agrees with existing literature [1, 4, 8, 13], due to a possible gender bias in hospitalization or due to genetic patterns of inheritance preferentially affecting males [15]. A high degree of consanguinity (26%) also resembles other studies [4, 19]. Significantly higher incidence of GDD and IEM occur in children born of consanguineous marriages. IEMs are mostly inherited in an autosomal recessive manner, which is seen with consanguineous marriages. Other Indian studies involving children with developmental delay reported seizures as a dominant symptom occurring in 42.9% and 90% children respectively, mostly generalized type [4, 15]. About half the children had hearing abnormalities and one fifth had vision abnormalities in one study [9], while another study reported visual abnormalities in 26.4% and hearing disturbances in 16.9% children [15].

In a study by Papavasiliou et al., a thorough physical examination revealed that 25% children had abnormalities [9]. These were mostly seen in the cardiovascular, genito-urinary, and musculoskeletal systems. Dysmorphism was noted in 19.5% children and abnormal neurological examination findings were noted in 56.8% [9]. Microcephaly was reported in up to 55% children in other studies [4, 15].

The positive yield of metabolic testing was 0.25 to 65% of diagnoses in GDD in the reported literature [4, 7–9, 16, 17, 20]. Such a wide range might be due to the small sample size, wide range of symptomatology and the hospital level at which the patient presented to. In the present study, the yield of initial metabolic tests was 26.4% and that of TMS and GC-MS tests was 20.7%. A higher yield was found in the present study, when compared to existing literature probably because it was conducted at a tertiary care hospital, which was the final point of referral with more complete evaluation and resources for the study participants. The detection of metabolic problems would definitely improve the outcome of these developmentally challenged children. Hence it would be recommended that physicians should consider metabolic evaluations positively when the etiology for GDD is unclear.

The limitations of the study include short study period of 18 mo with resultant small sample size. The longer study period would probably include the wider spectrum of yield and inclusion of rarer conditions. Another limitation was the economic constraints regarding the affordability of patients for Tandem Mass Spectroscopy and Gas Chromatography-Mass Spectrometry. The study did not collect the outcome of children in whom the vitamin supplements were given. The study was also limited to a single center.

## Conclusions

In children presenting with GDD of unclear etiology, a high degree of suspicion for metabolic disorders should be made. A comprehensive medical evaluation comprising detailed history, with special attention to consanguinity and positive family history; a meticulous physical examination with attention to growth and neurological examination, should prompt metabolic tests. The authors identified a probable metabolic disorder on initial metabolic tests in 26.4% of participants. TMS-GCMS testing confirmed a metabolic disorder in 20.7% of participants. Metabolic tests have their own role in such children. Metabolic evaluation is complementary to DNA testing in today’s time.
